# Supplementary material for: A foundation model for generalizable disease detection from retinal images
Source: Nature. 2023 Sep 13;622(7981):156–63. doi: 10.1038/s41586-023-06555-x (PMC10550819; doi:10.1038/s41586-023-06555-x)
Supplement: Supplementary file 1 — Supplementary Methods and Figs. 1–6. Figures 1–5 show the data flowcharts for ocular disease prognosis and systemic disease prediction. Figure 6 illustrates the architecture of RETFound. [file 41586_2023_6555_MOESM1_ESM.pdf]

nature portfolio

https://doi.org/10.1038/s41586-023-06555-x

Supplementary information

A foundation model for generalizable disease detection from retinal images

In the format provided by the authors and unedited

Supplementary Method Figures

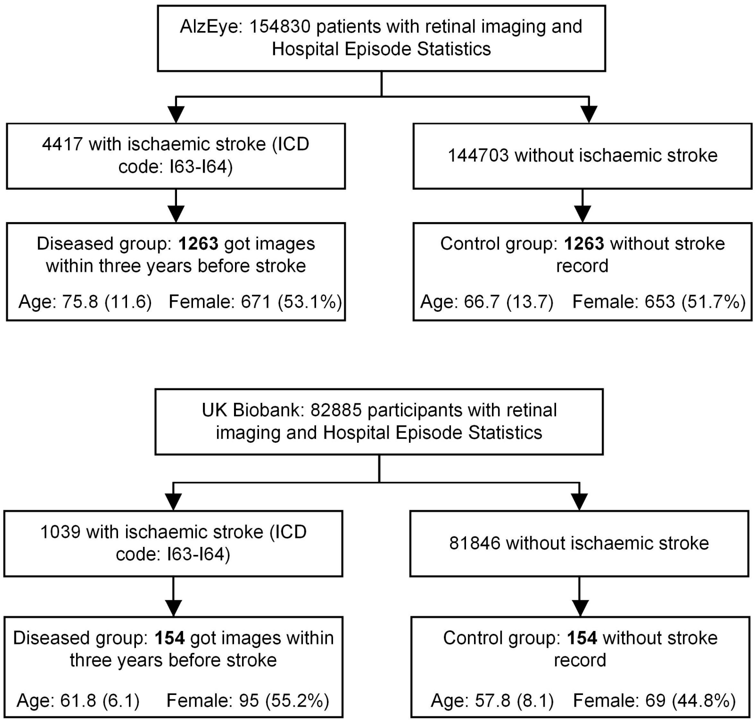

Supplementary Fig. 1. Flowchart of curating MEH-AlzEye and UK Biobank data for 3-year prediction of ischaemic stroke. MEH-AlzEye data is for fine-tuning SSL-based foundation model and internal evaluation, while UK Biobank is for external evaluation.

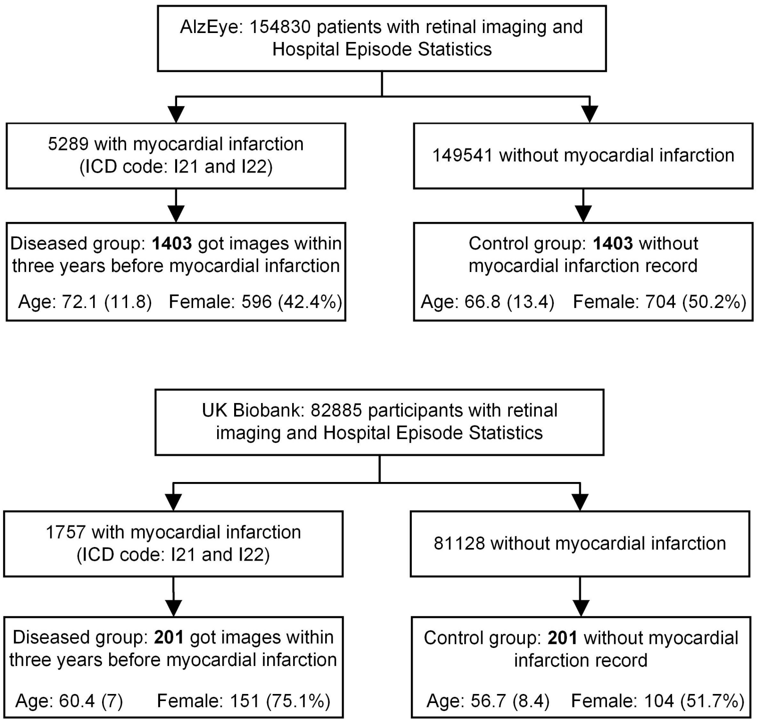

Supplementary Fig. 2. Flowchart of curating MEH-AlzEye and UK Biobank data for 3-year prediction of myocardial infarction. MEH-AlzEye data is for fine-tuning SSL-based foundation model and internal evaluation, while UK Biobank is for external evaluation.

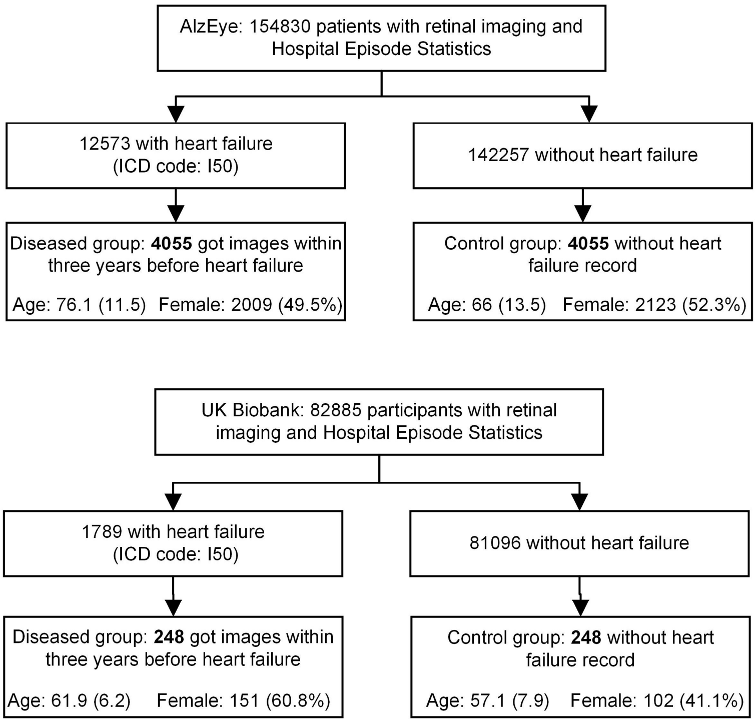

Supplementary Fig. 3. Flowchart of curating MEH-AlzEye and UK Biobank data for 3-year prediction of heart failure. MEH-AlzEye data is for fine-tuning SSL-based foundation model and internal evaluation, while UK Biobank is for external evaluation.

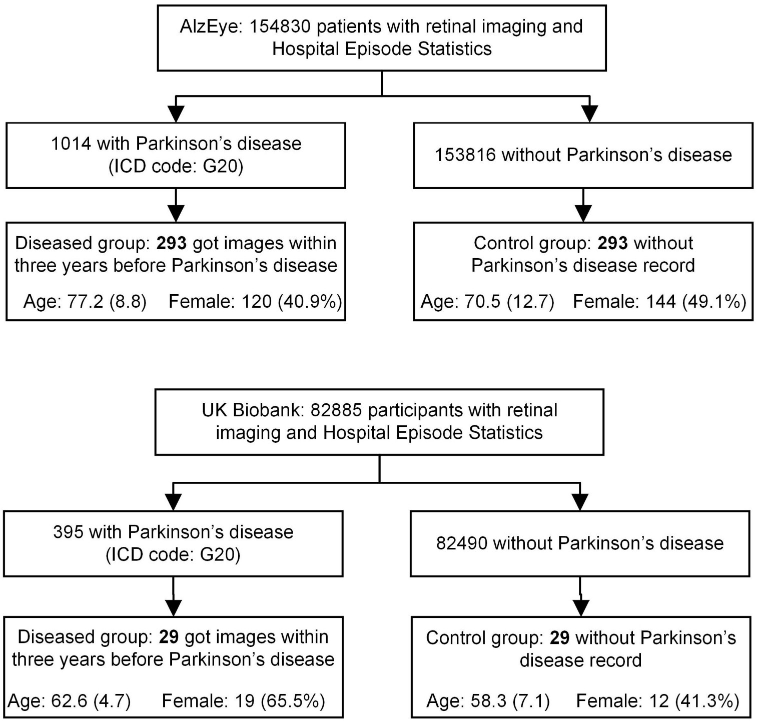

Supplementary Fig. 4. Flowchart of curating MEH-AlzEye and UK Biobank data for 3-year prediction of parkinson's disease. MEH-AlzEye data is for fine-tuning SSL-based foundation model and internal evaluation, while UK Biobank is for external evaluation.

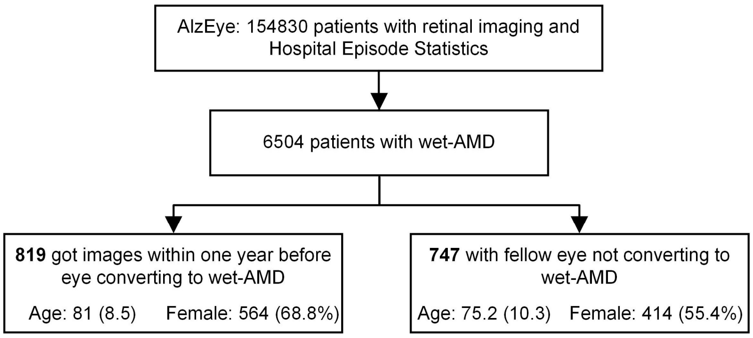

Supplementary Fig. 5. Flowchart of curating MEH-AlzEye for 1-year prognosis of fellow eye converting to wet-AMD. MEH-AlzEye data is for fine-tuning SSL-based foundation model and evaluation.

a

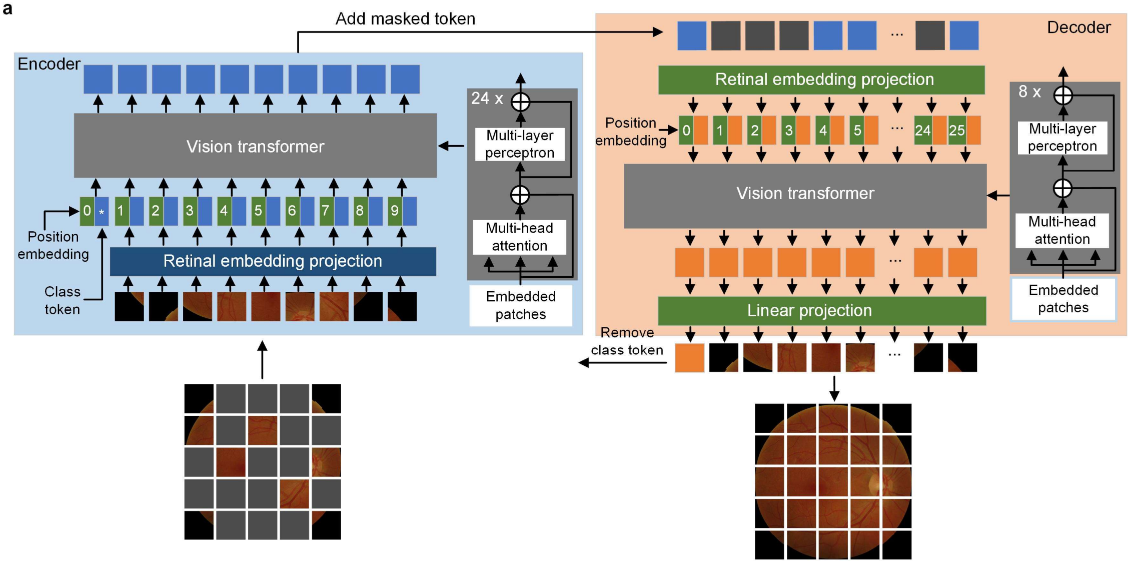

b

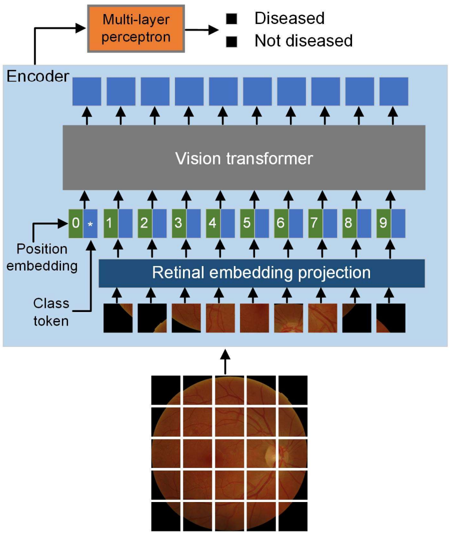

Supplementary Fig. 6. RETFound architecture. For self-supervised learning (a), a large vision Transformer-based encoder and a small vision Transformer-based decoder are included. The encoder takes unmasked patches as input and outputs high-level features. The decoder inserts dummy features as masked tokens and takes all features as input. The output of the decoder is the reconstructed patches for the original images. For fine-tuning the model to downstream tasks (b), only the encoder is needed. The encoder embeds the image patches and extracts the high-level features, which are used for classification and prediction after a multi-layer perceptron.
