# Supplementary material for: A foundation model for generalizable disease detection from retinal images
Source: Nature. 2023 Sep 13;622(7981):156–63. doi: 10.1038/s41586-023-06555-x (PMC10550819; doi:10.1038/s41586-023-06555-x)
Supplement: Supplementary file 2 — Reporting Summary [file 41586_2023_6555_MOESM2_ESM.pdf]

nature portfolio

Corresponding author(s): Pearse A. Keane, Yukun Zhou

Last updated by author(s): Aug 29, 2023

Reporting Summary

Nature Portfolio wishes to improve the reproducibility of the work that we publish. This form provides structure for consistency and transparency in reporting. For further information on Nature Portfolio policies, see our Editorial Policies and the Editorial Policy Checklist.

Statistics

For all statistical analyses, confirm that the following items are present in the figure legend, table legend, main text, or Methods section.

n/a	Confirmed	
☐	☒	The exact sample size (*n*) for each experimental group/condition, given as a discrete number and unit of measurement
☐	☒	A statement on whether measurements were taken from distinct samples or whether the same sample was measured repeatedly
☐	☒	The statistical test(s) used AND whether they are one- or two-sided<br>*Only common tests should be described solely by name; describe more complex techniques in the Methods section.*
☒	☐	A description of all covariates tested
☒	☐	A description of any assumptions or corrections, such as tests of normality and adjustment for multiple comparisons
☐	☒	A full description of the statistical parameters including central tendency (e.g. means) or other basic estimates (e.g. regression coefficient) AND variation (e.g. standard deviation) or associated estimates of uncertainty (e.g. confidence intervals)
☐	☒	For null hypothesis testing, the test statistic (e.g. *F*, *t*, *r*) with confidence intervals, effect sizes, degrees of freedom and *P* value noted<br>*Give P values as exact values whenever suitable.*
☒	☐	For Bayesian analysis, information on the choice of priors and Markov chain Monte Carlo settings
☒	☐	For hierarchical and complex designs, identification of the appropriate level for tests and full reporting of outcomes
☒	☐	Estimates of effect sizes (e.g. Cohen's *d*, Pearson's *r*), indicating how they were calculated

Our web collection on statistics for biologists contains articles on many of the points above.

Software and code

Policy information about availability of computer code

Data collection	The code used to train, fine-tune, and evaluate RETFound from Yukun Zhou is available at https://github.com/rmaphoh/RETFound_MAE which bases on PyTorch. Additionally, a Keras version implemented by Yuka Kihara is available at https://github.com/uw-biomedical-ml/RETFound_MAE. Please note that the reported results are obtained from PyTorch models. Image data was extracted from Dicom files with Pydicom v2.3.0 (https://pydicom.github.io). Images were processed with automated retinal image analysis tool AutoMorph v1.0 (https://github.com/rmaphoh/AutoMorph).
Data analysis	Data was analysed with Python v3.6 (https://www.python.org/), NumPy v1.19.5 (https://github.com/numpy/numpy), SciPy v1.5.4 (https://www.scipy.org/), seaborn v0.12.0 (https://github.com/mwaskom/seaborn), Matplotlib v3.6.1 (https://github.com/matplotlib/matplotlib), pandas v1.5.0 (https://github.com/pandas-dev/pandas), Scikit-Learn v1.1.3 (https://scikit-learn.org/stable), Pillow v9.2.0 (https://pypi.org/project/Pillow). Heatmaps were generated with RELPROP (https://github.com/hila-chefer/Transformer-Explainability).

For manuscripts utilizing custom algorithms or software that are central to the research but not yet described in published literature, software must be made available to editors and reviewers. We strongly encourage code deposition in a community repository (e.g. GitHub). See the Nature Portfolio guidelines for submitting code & software for further information.

Data

Policy information about availability of data

All manuscripts must include a data availability statement. This statement should provide the following information, where applicable:

- Accession codes, unique identifiers, or web links for publicly available datasets
- A description of any restrictions on data availability
- For clinical datasets or third party data, please ensure that the statement adheres to our policy

The MIDAS dataset consists of routinely collected healthcare data. Due to its sensitive nature and the risk of reidentification, the dataset is subject to controlled access via a structured application process. Data access enquiries may be made to enquiries@insight.hdrhub.org and we will aim to respond within two weeks. Further details about the data request pipeline may be found on the INSIGHT Health Data Research Hub website https://www.insight.hdrhub.org. The AlzEye dataset is subject to the contractual restrictions of the data sharing agreements between National Health Service Digital, Moorfields Eye Hospital and University College London and are not available for access beyond the AlzEye research team. National and international collaborations are welcomed though restrictions on access to the cohort mean that only the AlzEye researchers can directly analyse individual-level systemic health data. More details can be found at https://readingcentre.org/studies/artificial_intelligence/alzeye. UK Biobank data is available at https://www.ukbiobank.ac.uk/.

Data for ocular disease experiments are publicly available online and can be accessed via the links: IDRID (https://ieee-dataport.org/open-access/indian-diabetic-retinopathy-image-dataset-idrid), MESSIDOR-2 (https://www.adcis.net/en/third-party/messidor2/), APTOS-2019 (https://www.kaggle.com/competitions/aptos2019-blindness-detection/data), PAPILA (https://figshare.com/articles/dataset/PAPILA/14798004/1), Glaucoma Fundus (https://dataverse.harvard.edu/dataset.xhtml?persistentId=doi:10.7910/DVN/1YRRAC), JSIEC (https://zenodo.org/record/3477553), Retina (https://www.kaggle.com/datasets/jr2ngb/cataractdataset), OCTID (https://borealisdata.ca/dataverse/OCTID).

Human research participants

Policy information about studies involving human research participants and Sex and Gender in Research.

Reporting on sex and gender	Biological sex information for MEH-MIDAS and MEH-AlzEye was collected via self-report. MEH-MIDAS includes 37,401 patients (16,429 female, 20,966 male, and 6 unknown) and MEH-AlzEye includes 353,157 patients (190,494 female and 162,663 male). Experiments were conducted both on female and male. We used all MEH-MIDAS data to develop RETFound models and subsets of MEH-AlzEye for downstream validation (detailed in Supplementary Table 2).
Population characteristics	MEH-MIDAS is a retrospective dataset which includes the complete ocular imaging records of 37,401 patients with diabetes who were seen at Moorfields Eye Hospital, London, United Kingdom between 2000 and 2022. The age distribution has a mean value of 64.5 and standard deviation of 13.3. The ethnicity distributes diversly: British (13.7%), Indian (14.9%), Caribbean (5.2%), African (3.9%), other ethnicity (37.9%), not stated (24.4%). MEH-MIDAS includes various imaging devices, such as topcon 3DOCT-2000SA (Topcon), CLARUS (ZEISS), and Triton (Topcon).<br><br>MEH-AlzEye is a retrospective cohort study linking ophthalmic data of 353,157 patients, who attended Moorfields Eye Hospital between 2008 and 2018, with systemic health data from hospital admissions across the whole of England. Systemic health data are derived from Hospital Episode Statistics (HES) data relating to admitted patient care (APC), with a focus on cardiovascular disease and all-cause dementia. More details can be found in the method section. Selections of study cohort were shown in Supplementary Figure 2-6 and characteristics were listed in Supplementary Table 2.<br><br>The UK Biobank includes 502,665 UK residents aged between 40 and 69 years who are registered with the National Health Service. Among all participants, 82,885 get CFP and OCT examinations and a total of 171,500 retinal images are collected. Selections of study cohort were shown in Supplementary Figure 2-6 and characteristics were listed in Supplementary Table 2.
Recruitment	MEH-MIDAS is a retrospective dataset which includes the complete ocular imaging records of 37,401 patients with diabetes who were seen at Moorfields Eye Hospital, London, United Kingdom between 2000 and 2022. MEH-AlzEye is a retrospective cohort study linking ophthalmic data of 353,157 patients who attended Moorfields Eye Hospital between 2008 and 2018.
Ethics oversight	This study involves human participants and was approved by the London-Central Research Ethics Committee (18/LO/1163, approved 01/08/2018), Advanced statistical modelling of multimodal data of genetic and acquired retinal diseases (20/HRA/2158, approved 05/05/2020), and the Confidential Advisory Group for Section 251 support (18/CAG/0111, approved 13/09/2018). The National Health Service Health Research Authority gave final approval on 13 September 2018. Moorfields Eye Hospital NHS Foundation Trust validated the de-identifications. Only de-identified retrospective data was used for research, without the active involvement of patients.

Note that full information on the approval of the study protocol must also be provided in the manuscript.

Field-specific reporting

Please select the one below that is the best fit for your research. If you are not sure, read the appropriate sections before making your selection.

☒ Life sciences ☐ Behavioural & social sciences ☐ Ecological, evolutionary & environmental sciences

For a reference copy of the document with all sections, see nature.com/documents/nr-reporting-summary-flat.pdf

Life sciences study design

All studies must disclose on these points even when the disclosure is negative.

Sample size	Data for developing RETFound model was from Moorflelds Diabetic imAge dataSet (MEH-MIDAS) and public data (totalling 904,170 CFPs and 736,442 OCTs). Data for ocular disease diagnosis were from public datasets, detailed in Supplementary Table 1. Data for systemic disease prediction were from Moorfields AlzEye project and selected cohorts were introduced in Supplementary Table 2. Datasets were chosen based on the availability of labels that would permit external validation of the different fine-tuned RETFound models, which is dependent on the specific clinical task being evaluated. The chosen external validation datasets were deemed to be suitable based on their parameters, which are summarised Supplementary Information Table 1 Dataset characteristics. Formal sample size calculations were not performed due to the lack of established methods when applied to machine-learning classification studies.
Data exclusions	Data failed image processing with AutoMorph were excluded. Data without systemic health labels were excluded. For more details please refer to the method section.
Replication	All patients were randomly selected and were not correlated in any way. The replication of experiment results were confirmed in 5 times with 5 different random seeds.
Randomization	The training/validation/testing data for downstream tasks were randomly splitted in ratio of 55%:15%:30%. For each patient, we only included the left eye data from one visit to avoid potential bias by inconsistent individual visits.
Blinding	When assigning patients randomly to training, validation and testing groups investigators were blinded to patient covariates and all features in the dataset not required to perform the research.

Reporting for specific materials, systems and methods

We require information from authors about some types of materials, experimental systems and methods used in many studies. Here, indicate whether each material, system or method listed is relevant to your study. If you are not sure if a list item applies to your research, read the appropriate section before selecting a response.

Materials & experimental systems

n/a	Involved in the study	
☒	☐	Antibodies
☒	☐	Eukaryotic cell lines
☒	☐	Palaeontology and archaeology
☒	☐	Animals and other organisms
☒	☐	Clinical data
☒	☐	Dual use research of concern

Methods

n/a	Involved in the study	
☒	☐	ChIP-seq
☒	☐	Flow cytometry
☒	☐	MRI-based neuroimaging
